# The new paradigm of hepatitis C therapy: integration of oral therapies into best practices

**DOI:** 10.1111/jvh.12173

**Published:** 2013-10-07

**Authors:** N H Afdhal, S Zeuzem, R T Schooley, D L Thomas, J W Ward, A H Litwin, H Razavi, L Castera, T Poynard, A Muir, S H Mehta, L Dee, C Graham, D R Church, A H Talal, M S Sulkowski, I M for the New Paradigm of HCV Therapy Meeting Participants Jacobson

**Affiliations:** 1 Division of Gastroenterology and Hepatology, Beth Israel Deaconess Medical Center, Harvard Medical SchoolBoston, MA, USA; 2 Department of Medicine, J.W. Goethe University HospitalFrankfurt, Germany; 3 Division of Infectious Diseases, San Diego School of Medicine, University of CaliforniaLa Jolla, CA, USA; 4 Division of Infectious Diseases, Johns Hopkins University School of MedicineBaltimore, MD, USA; 5 Division of Viral Hepatitis, Centers for Disease Control and PreventionAtlanta, GA, USA; 6 Departments of Medicine and Psychiatry and Behavioral Sciences, Montefiore Medical Center, Albert Einstein College of MedicineBronx, NY, USA; 7 Center for Disease AnalysisLouisville, CO, USA; 8 Service d'Hepatologie, Hopital Beaujon, Assistance Publique Hopitaux de ParisClichy, France; 9 Service d'Hepatologie, Groupe Hospitalier Pitie-SalpetriereParis, France; 10 Gastroenterology and Hepatology Research Group, Duke Clinical Research InstituteDurham, NC, USA; 11 Department of Epidemiology, Johns Hopkins Bloomberg School of Public HealthBaltimore, MD, USA; 12 Fair Pricing Coalition and AIDS Action BaltimoreBaltimore, MD, USA; 13 Division of Infectious Disease, Beth Israel Deaconess Medical CenterBoston, MA, USA; 14 Massachusetts Department of Public Health, Bureau of Infectious DiseaseBoston, MA, USA; 15 Division of Gastroenterology and Hepatology, Department of Internal Medicine, University at BuffaloBuffalo, NY, USA; 16 Department of Medicine, Johns Hopkins University School of MedicineBaltimore, MD, USA; 17 Division of Gastroenterology and Hepatology, Weill Cornell Medical CollegeNew York, NY, USA

**Keywords:** diagnosis, directly acting antiviral agents, health services, hepatitis C, pharmacoeconomics

## Abstract

SUMMARY. Emerging data indicate that all-oral antiviral treatments for chronic hepatitis C virus (HCV) will become a reality in the near future. In replacing interferon-based therapies, all-oral regimens are expected to be more tolerable, more effective, shorter in duration and simpler to administer. Coinciding with new treatment options are novel methodologies for disease screening and staging, which create the possibility of more timely care and treatment. Assessments of histologic damage typically are performed using liver biopsy, yet noninvasive assessments of histologic damage have become the norm in some European countries and are becoming more widespread in the United States. Also in place are new Centers for Disease Control and Prevention (CDC) initiatives to simplify testing, improve provider and patient awareness and expand recommendations for HCV screening beyond risk-based strategies. Issued in 2012, the CDC recommendations aim to increase HCV testing among those with the greatest HCV burden in the United States by recommending one-time testing for all persons born during 1945–1965. In 2013, the United States Preventive Services Task Force adopted similar recommendations for risk-based and birth-cohort-based testing. Taken together, the developments in screening, diagnosis and treatment will likely increase demand for therapy and stimulate a shift in delivery of care related to chronic HCV, with increased involvement of primary care and infectious disease specialists. Yet even in this new era of therapy, barriers to curing patients of HCV will exist. Overcoming such barriers will require novel, integrative strategies and investment of resources at local, regional and national levels.

## Introduction

The treatment landscape for hepatitis C is in flux. From 2002 to 2011, the standard of care treatment for chronic infection with hepatitis C virus (HCV) was 24 or 48 weeks of therapy with pegylated interferon-alfa (PEG-IFN) and ribavirin (RBV). For patients with genotype 1 virus, the likelihood of achieving a sustained virological response (SVR), defined as having undetectable serum HCV RNA at 24 weeks after cessation of treatment, was only 40–50% after 48 weeks of therapy. In 2011, the HCV protease inhibitors telaprevir and boceprevir entered the market to be used in combination with PEG-IFN and RBV for genotype 1 HCV infection. The protease inhibitors increase the likelihood of SVR to 67–75% in treatment-naïve patients with genotype 1 HCV 1–4. However, adding a protease inhibitor to a PEG-IFN backbone, which is itself difficult to tolerate, has increased the potential for toxicity and has placed a resource-intensive burden on treating physicians. In addition, the triple therapy regimens have limited efficacy in treatment-experienced null responders 5.

Several directly acting antiviral agents are being evaluated for their potential use in combination with either RBV or other antivirals of different classes. In early 2013, a small study of the nucleotide sofosbuvir in combination with RBV was reported, and among 25 treatment-naïve, HCV genotype 1 patients, 21 (84%) had an SVR after 12 weeks of therapy 6. In another small study reported in early 2013, a total of 31 of 33 (94%) previously treatment-naïve genotype 1 patients were HCV RNA negative 12 weeks after cessation of therapy with the NS3 protease inhibitor ABT-450, combined with low-dose ritonavir, the non-nucleoside NS5B polymerase inhibitor ABT-333 and ribavirin 7. In phase 3 studies of sofosbuvir with RBV, SVR rates have been as high as 78% in HCV genotype 2 and 3 patients 8, and it is anticipated that in the United States, all-oral combination therapies will be available for HCV genotype 2 or 3 patients by 2014. By 2015, regimens including only directly acting antivirals are expected to be available for persons with any HCV genotype.

At this time of new treatment opportunities, novel changes have been made to improve the methods by which extent of liver disease is diagnosed. Assessment of the extent of histologic damage, an important component of patient evaluation, has been traditionally carried out by liver biopsy. Noninvasive assessments of histologic damage such as elastography have become the norm in several European countries and are becoming more frequently used in the United States. Strategies for HCV screening also have been revised. To improve the identification of persons living with chronic HCV infection, the U.S. Centers for Disease Control and Prevention (CDC) expanded its risk-based approach to HCV testing, publishing a recommendation in 2012 that all persons born during 1945–1965 receive one-time testing for HCV. In 2013, the United States Preventive Services Task Force (USPSTF) adopted similar recommendations for risk-based and birth-cohort-based testing. Growing evidence suggests that in the United States, HCV infections are rapidly increasing among persons aged 15–24 primarily because of injection drug use 9. This trend suggests that screening efforts should also ensure that young injection drug users are tested and engaged in care.

The 2010 Patient Protection and Affordable Care Act (ACA) will expand opportunities for persons to purchase health insurance and have access to hepatitis C testing, care and treatment. The ACA will facilitate implementation of HCV testing because it requires nongrandfathered private health plans to cover clinical preventive services given an A or B grade by the USPSTF without cost-sharing and provides incentives for Medicaid programmes to cover these services. By prohibiting insurance companies from declining to sell or renew policies because of pre-existing conditions such as hepatitis C, ACA will help more patients access HCV care and treatment services 10.

Improvements in therapies, diagnostic techniques and screening for HCV will create a new era for HCV treatment. Although the exact effects these changes will have on the future landscape of HCV care cannot be elucidated, certain outcomes are likely. For instance, as methods for diagnosis of liver disease and treatment of HCV become simpler, safer and more effective, primary healthcare providers may manage greater numbers of HCV-infected patients. This expansion into primary care may become necessary if the number of patients undergoing treatment increases because of screening efforts and improved prospects for treatment success for regimens containing all directly acting antivirals. Also subject to change are pricing and reimbursement models, as well as the pharmacoeconomics of curing HCV.

To discuss the new paradigm of HCV therapy, representatives from leading academic medical centres, government agencies, insurance providers and the pharmaceutical and biotechnology industries met in Boston, Massachusetts, USA, on March 22 and 23, 2013. The focus of the meeting, or Think Tank, was to predict how shifts in HCV screening, diagnosis and treatment will affect access to and delivery of care; identify barriers to treating HCV; discuss successful strategies for identifying and treating patients; and discuss the pharmacoeconomics of treatment for patients, providers, pharmaceutical companies and healthcare payers. Here, we describe the current challenges and opportunities for curing HCV in the forthcoming era.

## Current Evolution of All-Oral Therapies for HCV

Arrays of IFN-free regimens for treating HCV are currently in the later stages of clinical development. At scientific meetings, data have been presented from phase 2 and 3 studies of various all-oral regimens. The results of individual studies will not be described in great detail here but are summarized in Table 1. The more promising regimens have the following characteristics: a strong safety profile, SVR rates approaching or even exceeding 90%, minimal pill burden and minimal potential for drug–drug interactions.

**Table 1:** Reported results for all-oral therapies for hepatitis C virus in clinical development

	No. patients	Duration, weeks	SVR rates	Reference
Treatment-naïve patients				
Genotype 1 (1a or 1b)				
ABT-450/r + ABT-333 + RBV	33	12	94% SVR12	Poordad *et al*. 7
ABT-450/r + ABT-267 + ABT-333 + RBV	80	8	88% SVR12	Kowdley *et al*. 11
ABT-450/r + ABT-333 + RBV	41	12	85%	Kowdley *et al*. 11
ABT-450/r + ABT-267 + RBV	79	12	90%	Kowdley *et al*. 11
ABT-450/r + ABT-267 + ABT-333	79	12	87%	Kowdley *et al*. 11
ABT-450/r + ABT-267 + ABT-333 + RBV	79	12	98%	Kowdley *et al*. 11
Daclatasvir + Asunaprevir + BMS-791325	16	24	88%	Everson *et al*. 98
Daclatasvir + Asunaprevir + BMS-791325	16	12	94%	Everson *et al*. 98
Faldaprevir + Deleobuvir	46	28	39% SVR12	Zeuzem *et al*. 15
Faldaprevir + Deleobuvir + RBV	316	16, 28, or 40	52–69% SVR12	Zeuzem *et al*. 15
Mericitabine + Danoprevir + RBV	64	24	71% SVR12	Gane *et al*. 99
Sofosbuvir + RBV	25	12	84%	Gane *et al*. 6
Sofosbuvir + Daclatasvir	55	12 or 24	98% SVR4	Sulkowski *et al*. 14
Sofosbuvir + Daclatasvir + RBV	56	12 or 24	96% SVR4	Sulkowski *et al*. 14
Sofosbuvir + Ledipasvir + RBV	25	12	100% SVR12	Gane *et al*. 101
Genotype 2 or 3				
Sofosbuvir + RBV	10	12	100%	Gane *et al*. 6
Sofosbuvir	10	12	60%	Gane *et al*. 6
Sofosbuvir + RBV	253	12	67% SVR12	Gane *et al*. 100
Sofosbuvir + Daclatasvir	14	24	100%	Sulkowski *et al*. 14
Sofosbuvir + Daclatasvir + RBV	14	24	93%	Sulkowski *et al*. 14
Prior nonresponse				
Genotype 1 (1a or 1b)				
ABT-450/r + ABT-333 + RBV	17	12	47% SVR12	Poordad *et al*. 7
ABT-450/r + ABT-267 + RBV	45	12	89%	Kowdley *et al*. 11
ABT-450/r + ABT-267 + ABT-333 + RBV	45	12	93%	Kowdley *et al*. 11
Daclatasvir + Asunaprevir	11	24	36%	Lok *et al*. 102
Sofosbuvir + RBV	10	12	10%	Gane *et al*. 6
Sofosbuvir + Daclatasvir	21	12	100%	Sulkowski *et al*. 13
Sofosbuvir + Daclatasvir + RBV	20	12	95%	Sulkowski *et al*. 13
Sofosbuvir + Simeprevir + RBV	27	12	96% SVR8	Lawitz *et al*. 12
Sofosbuvir + Simeprevir	14	12	93% SVR8	Lawitz *et al*. 12
Sofosbuvir + Ledipasvir + RBV	10	12	100% SVR12	Gane *et al*. 101
Genotype 1b				
Daclatasvir + Asunaprevir	21	24	91%	Suzuki *et al*. 103
Genotype 2 or 3				
Sofosbuvir + RBV	201	12 or 16	SVR12 12 week: 50% 16 week: 73%	Jacobson *et al*. 8
IFN-ineligible or intolerant				
Genotype 1b				
Daclatasvir + Asunaprevir	22	24	64%	Suzuki *et al*. 103
Genotype 2 or 3				
Sofosbuvir + RBV	207	12	78% SVR12	Jacobson *et al*. 8

ABT-450/r, ritonavir-boosted ABT-450; IFN, interferon; RBV, ribavirin; SVR, viral negativity 24 weeks post-therapy.

SVR12, SVR8 and SVR4 refer to viral negativity at 12, 8 and 4 weeks post-therapy.

Several major conclusions and predictions regarding the future of all-oral therapies were discussed. A reasonable anticipation is that a genotype-specific, all-oral therapy for HCV genotypes 2 and 3 with sofosbuvir and ribavirin will be available by 2014. By 2015, genotype-1-specific therapies should follow, and these will comprise any of three regimens currently under development by AbbVie Pharmaceuticals, Bristol-Myers Squibb and Gilead Sciences (Table 1). True pangenotypic regimens will probably not be available until 2016 or 2017 and will require development of pangenotypic NS5A inhibitors and protease inhibitors that can be combined with each other and with nucleotide polymerase inhibitors. Some of these combinations are in phase 1 or early phase 2 studies across multiple genotypes.

Another major area of discussion was whether pretreatment and on-treatment predictors of response, including those used for PEG-IFN, can help predict response to all-oral therapies. Although there is evidence that many of these factors still predict response to relatively weak interferon-sparing regimens, more potent regimens, with SVR rates >90%, readily overcome the traditional obstacles seen with PEG-IFN. For example, in phase 2 studies of the Abbott multidrug regimen 11 or sofosbuvir plus simeprevir 12 or daclatasvir 13, prior interferon response was not strongly related to response to all-oral treatment. In fact, interferon null responders did just as well as naïve patients and had SVR rates in the 90% range. It is increasingly apparent that regimens consisting of potent agents that individually or cumulatively impose a high barrier to resistance attenuate or eliminate factors such as 1a/1b subtype, *IL28B* status, viral load, race, metabolic syndrome, obesity and age as major determinants of response. In addition, with potent directly acting antiviral combinations, nearly all patients are negative within 4 weeks, which means the traditional strategy of using virologic response at week 4 or 12 to determine the duration of treatment may be moot. The presence of cirrhosis, which often excludes patients from early phase trials, may yet be a differentiating factor in SVR rates, but this remains to be further determined for genotype 1, and as with other factors, presence of cirrhosis can probably be overcome by a sufficiently potent regimen or longer treatment duration. In studies of sofosbuvir and RBV in patients with HCV genotypes 2 or 3, cirrhosis was a significant negative predictor of response for treatment-naïve patients with HCV genotype 3 and for prior treatment-failure patients with either genotype 2 or 3, but these studies only included 1 potent directly acting antiviral. The effect of portal hypertension and hepatocellular dysfunction (Child's class B and C) on SVR in patients with more advanced liver disease remains an area requiring additional investigation.

The final major questions for discussion encompassed the need for RBV and duration of therapy, which are in some ways connected. As with the pretreatment predictors, ribavirin use and treatment duration appear to matter with relatively weak regimens but may not with sufficiently potent combinations. In studies of the polymerase inhibitor sofosbuvir with either the NS5A inhibitor daclatasvir 14 or the protease inhibitor simeprevir 12, SVR rates were independent of RBV use. However, in a study combining the protease inhibitor faldaprevir and the non-nucleoside polymerase inhibitor deleobuvir, omitting RBV resulted in a marked reduction in efficacy in genotype 1a patients 15. And for HCV genotype 1a patients in the phase 2 AVIATOR trial 11, the removal of RBV from a regimen containing the ritonavir-boosted protease inhibitor ABT-450/r, the NS5A inhibitor ABT-267 and the non-nucleoside polymerase inhibitor ABT-333 resulted in a 10% loss of efficacy. The optimal duration of therapy remains unknown, but with potent regimens, 12 weeks is probably the maximum required for most patients (with the potential exception for patients with advanced cirrhosis). Eight-week treatment regimens can be explored, although this may result in a moderate (∼10%) reduction in SVR 11 depending on the regimen.

For regimens containing only direct-acting antivirals, one could imagine a scenario where more potentially difficult-to-treat patients are distinguished from a potentially more easily treatable population. Difficult-to-treat patients may be best served by undergoing an individualized regimen under the care of a specialist. Individualized therapy could be based upon HCV genotype, fibrosis stage, comorbidities, concomitant medications or prior directly acting antiviral drug exposure. Populations of patients who may require individualized therapy but for whom evidence-based treatment data are limited include those with cirrhosis, including decompensated cirrhosis, HIV coinfection, renal failure, an organ transplant or other conditions resulting in being immunocompromised. In the future, it is possible that the population of HCV positive individuals with F0-2 histology will undergo treatment without further stratification such as via HCV genotype or *IL28B* polymorphisms, because SVR rates will likely be in the 90% range.

## Screening Strategies for HCV

In the United States, mortality associated with hepatitis C is on the rise and currently exceeds that for HIV 16. On the basis of survey data from 1999 to 2002, it has been estimated that 3.2 (2.7–3.9) million persons in the United States have chronic HCV infection 17. The strongest risk of HCV infection is a history of injection drug use 17. Of persons with chronic infection, 74% were born during the years 1945 through 1965 18.

In 1998, CDC recommended a risk-based approach to screening, with routine HCV testing for persons with risk factors including injection drug use, having received clotting factor concentrates produced before 1987, being on chronic haemodialysis, having persistently abnormal alanine aminotransferase levels, being a recipient of donated blood from a person who tested positive for HCV, or having received a blood transfusion or organ transplant before July 1992 19; in 1999, CDC recommended HCV testing for persons with HIV. The 2009 guidelines from the American Association for the Study of Liver Diseases (AASLD) 20 and the 2006 guidelines from the American College of Gastroenterology 21 also recommend screening in high-risk patients. However, risk-based screening strategies can be limited either by clinician reluctance to ask about risk factors or by patient unawareness or reluctance to disclose risk behaviours. As a result, use of risk-based strategies alone has resulted in a large proportion of infected persons remaining undiagnosed; in the United States, various estimates indicate that 45–85% of persons with HCV are unaware of their infection status 22–25. To augment risk-based screening, in 2012 CDC published a recommendation for one-time testing without prior ascertainment of HCV risk for persons born during 1945–1965 25. This birth-cohort approach was designed to both target persons with the highest prevalence of HCV infection and remove any behavioural stigma from screening. In the state of New York, legislation requiring all patients born within the birth-cohort period to be offered hepatitis C screening when they visit healthcare providers has passed the legislature and is awaiting the governor's approval.

It has been estimated that with implementation of the birth-cohort screening strategy, 121 000 deaths from HCV will be averted 26. In recognition of these and other data 27–28, in June 2013 the USPSTF issued a final recommendation regarding HCV testing, assigning a Grade B to two recommendations: screening for HCV infection in persons at high risk for infection and one-time HCV screening for adults born between 1945 and 1965 29. A USPSTF Grade B designation expands access to clinical preventive services.

For HCV screening to become widely adopted in diverse clinical settings providing care for persons at risk for HCV infection, efforts are needed at local, regional and national levels. Approximately 79 million persons were born during 1945–1965 (the Baby Boom Generation), making birth-cohort based screening a daunting task. However, since the release of the CDC recommendations, multiple independent studies of HCV testing have shown birth-cohort-based approaches superior to risk-based strategies alone 30–31. To increase the numbers of HCV-infected persons aware of their infection, CDC is implementing a national multimedia campaign, Know More Hepatitis, that includes education for consumers and healthcare providers (http://www.cdc.gov/knowmorehepatitis/). Specific initiatives include messaging on airport dioramas and billboards in cities such as Atlanta, Washington, DC, Salt Lake City, Orlando and Las Vegas; online medical education for health professionals (http://depts.washington.edu/hepstudy/hepC/); and the launch of an annual National Hepatitis Testing Day observed on May 19th.

Centers for Disease Control and Prevention is also conducting demonstration projects to evaluate the implementation of risk-based and birth-cohort strategies for HCV strategies in over 25 clinical settings. For example, the Hepatitis C Assessment and Testing Project in New York City evaluated community-based screening interventions in three urban primary care clinics 32. Both risk-based and birth-cohort-based interventions were associated with an increased proportion of patients tested for HCV. Both risk-based and birth-cohort HCV screening approaches can be integrated within electronic medical records.

The new HCV screening recommendations are expected to increase demand for testing to detect current HCV infection. To meet this demand, CDC recently simplified the HCV testing sequence 33. Patients should first be tested for HCV antibody. Patients who are reactive for HCV antibody should next be tested with an FDA-approved nucleic acid testing assay for the detection of HCV RNA indicative of current HCV infection. Rapid tests for HCV antibody allow access to HCV testing in settings lacking laboratory-based diagnostic services. Rapid tests for HCV antibody detection include OraQuick 34, which is approved by the United States Food and Drug Administration (FDA), as well as Chembio 34–35, MedMira 34–35 and mBio, which are under development.

## HCV Diagnostic Testing and Disease Staging

It is likely that the rapid improvements in treatment efficacy and tolerability anticipated with interferon-sparing regimens will also transform our approach to disease staging. The historic low efficacy and safety of interferon-based regimens led to the recommendation for liver staging to determine whether the benefits of treatment would outweigh the risks. For most patients, this required that there be more than just portal fibrosis. Liver biopsy was considered the best test for this purpose, but the procedure is costly, invasive and in a small minority of cases can result in complications such as significant bleeding, organ puncture, or death 36. And the accuracy in staging disease is often compromised by variability in tissue sampling or in interobserver or intra-observer histopathological scoring.

As treatment efficacy for genotype 2 and 3 infections improved, biopsy was no longer routinely recommended to justify treatment necessity 20. Likewise, continued improvements in treatment efficacy and safety for all genotypes will change the primary goal of staging from justification of treatment benefit to identification of persons with cirrhosis or bridging fibrosis because they may need longer treatment courses and require hepatocellular carcinoma screening and portal hypertension management. Accordingly, the most important characteristic of a staging test is the negative predictive value for detection of cirrhosis.

Noninvasive methods of assessing histology are becoming more widely used. These include measurement of serum biomarkers of fibrosis and measurement of liver stiffness through elastography (Table 2). The noninvasive methods have practically no complications and can be performed repeatedly to dynamically monitor progression of fibrosis. The rate of adoption of noninvasive diagnostic tests for liver fibrosis differs between international regions, and the United States lags behind Europe in this regard. The 2012 European Association for the Study of the Liver (EASL) guidelines for treating chronic hepatitis C suggest that while liver biopsy is still regarded as the reference method for grading inflammation and staging fibrosis, transient elastography can be used to assess liver fibrosis, and noninvasive serum markers are recommended for detecting significant fibrosis (METAVIR score F2-F4) 37.

**Table 2:** FibroScan and FibroSure* for diagnosis of cirrhosis

	FibroScan	FibroSure
AUROC, mean (95% CI)	0.94 (0.93–0.95) 41	0.86 (0.71–0.92) 38
Sensitivity (95% CI)	0.83 (0.79–0.86) 104	0.85 38
Specificity (95% CI)	0.89 (0.87–0.91) 104	0.81 38
Advantages	Evaluates a genuine property of the liver	Good reproducibility
	High performance for cirrhosis	High applicability (>95%)
	User-friendly, point-of-contact test	
	Good reproducibility	
Disadvantages	Decreased performance in obese patients	Nonspecific of the liver
	Applicability lower than serum biomarkers: failure in 3% of cases and unreliable results in 16% (obesity, ascites, limited operator experience)	
	Requires a dedicated device	
	Inflammation, extra-hepatic cholestatis, and right heart failure can provide false positive results	

* Known as FibroTest in Europe.

In 2004, the biomarker assay FibroSure (named FibroTest in Europe) was launched in the United States for assessing fibrosis and necroinflammatory activity. The assay, which can only be performed in validated laboratories, predicts a histology score on the basis of patient age, sex and results for serum haptoglobin, α2-macroglobulin, apolipoprotein A1, γ-glutamyltransferase and bilirubin analyses 38. In a review of 25 studies in chronic HCV, FibroTest had an AUROC of 0.79 (0.70–0.89) for diagnosis of significant fibrosis (*F* ≥ 2) and 0.86 (0.71–0.92) for liver cirrhosis 38.

If the birth-cohort screening to enhance diagnosis of HCV infection is fully implemented, it has been estimated that as many as 800 000 additional cases of HCV infection would be identified 26. Should there be such a large-scale influx of newly diagnosed patients, tests of serum biomarkers will likely be a more practical approach to liver disease staging than one restricted to liver biopsy-based staging. In the United States, the FibroSure test costs approximately $250, which is a fraction of the cost of biopsy. The biomarker assay AST-to-Platelet Ratio Index (APRI) is not proprietary and costs no more than a routine blood draw and routine liver function tests. APRI is calculated as (AST/upper limit of normal range)/platelet count (10^9^/L) × 100. However, a recent large meta-analysis suggested that APRI can identify hepatitis C-related fibrosis with only a moderate degree of accuracy (AUROC of 0.77 for significant fibrosis and 0.80 for severe fibrosis) 39. Alternate *in vitro* diagnostic testing for liver fibrosis was subsequently developed, including FibroIndex and Forns index 38. For identifying cirrhosis, the age-platelet index, APRI and Hepascore have median AUROCs of 0.80 or greater (range 0.80–0.91) 38.

Transient elastography, using the FibroScan device (Echosens, Paris, France), is widely used in several European countries and has more recently been adopted in Asia and Canada. In April of 2013, FibroScan was approved by the FDA for use in the United States. The main limitation of FibroScan use in practice is its limited applicability (80%), mostly due to patient obesity or limited operator experience 40. Results of a meta-analysis suggest FibroScan is a reliable method for diagnosing significant fibrosis (AUROC = 0.84), severe fibrosis (0.89) and particularly cirrhosis (0.94) 41. However, for diagnosing significant fibrosis, a high variation of the AUROC was found depending on the type of underlying liver disease 41. When compared and validated externally in a multicenter prospective study, FibroScan outperformed serum biomarkers of fibrosis for the prediction of cirrhosis (AUROCS 0.89–90 *vs* 0.77–0.86) but had similar performance for the diagnosis of significant fibrosis 42. Both FibroScan and FibroTest have a prognostic value similar to liver biopsy for predicting complications and outcome of liver disease 43–44. Combining FibroScan with Fibrotest may increase diagnostic performance for significant fibrosis 45–46, and this approach has been recommended in the 2012 EASL Guidelines as first line evaluation of liver fibrosis in patients with chronic hepatitis C 37.

The Think Tank recognized that the goals of liver staging are changing and that there is an urgency to revise guidelines accordingly. The accurate exclusion of cirrhosis has been recommended as the most important role for HCV staging in clinical practice, and an algorithm has been proposed on how to best utilize noninvasive tests to achieve this goal 47.

## Barriers to Care and Strategies to Address Them

**Figure 1:**
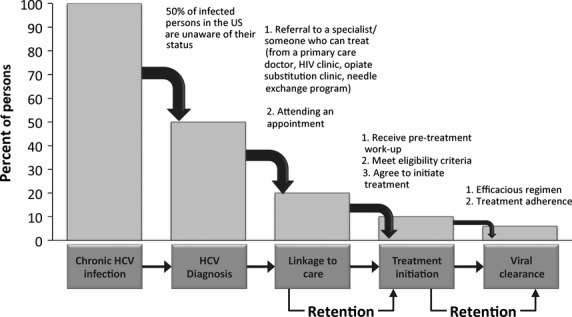
The hepatitis C care cascade. Among patients infected with hepatitis C virus, fewer than 10% are treated and cured. Barriers exist in screening methods, patient referral to appropriate providers, attending necessary appointments and initiating treatment 49–113.

Among persons infected with HCV, a substantial portion fails to progress towards a cure at every step of treatment, from recognition of disease to viral clearance (Fig. 1) 48. In the United States, at least half of those infected with HCV do not know their status 22–25. Among patients who are recognized as being positive for HCV antibody, it is estimated that fewer than half are linked to care 49. Failure to link to care represents both a lack of referral to a specialist for treatment and failure to attend the appointment. Even after being linked to proper caregivers, patients can fail to receive pretreatment work-up, meet eligibility criteria for treatment or agree to initiate treatment.

Reasons for these failures can be attributed to barriers at the level of patients, providers and the healthcare system itself. Patients can have limited access to health care, because of lack of or limited insurance 50, low health literacy or not having a usual source of medical care. They can also have competing health priorities, such as mental health issues 51 or comorbidities 52. Issues related to patient behaviours or environment, such as substance abuse 53–54, lack of drug treatment, lack of social support 55 or unstable employment or housing 56, may also limit uptake of treatment. Patients may also have limited knowledge of HCV and its treatment and may not perceive it as being something they need to worry about because the disease is largely asymptomatic 57. Finally, many patients fear the side effects of IFN-based regimens 57.

Primary care providers can have misconceptions about whom to screen, risk of progression of liver disease or ztherapy itself 58–59. Even specialists in liver disease may have limited experience treating HCV 60. Providers also can be selective about which patients they consider as good candidates for therapy and fail to recommend treatment because of concerns about nonadherence, drug use 61 or risk of re-infection.

Governments and payers play key roles in delivering HCV services; surveying infection, testing and treatment rates; and educating the public and as well as healthcare providers. Unfortunately, the United States has poorly developed surveillance systems, inadequate educational initiatives and fragmented viral hepatitis services 62. Also at issue are insufficient numbers of providers who can and are willing to treat HCV 63 and insufficient resources for case managers, navigators and social workers.

Training community-based healthcare providers to treat HCV may become a key method for broadening access to cure. Community-based health centres often have advantages of being culturally appropriate and accessible to patients in both urban and rural areas. In these settings, ongoing relationships with providers may establish trust and an avenue for communication. The Extension for Community Healthcare Outcomes model was developed as a means of using video-conferencing to train primary care providers through interactions with specialists to treat complex diseases, such as HCV. In New Mexico, the programme was successful in generating rates of SVR at 21 sites in rural areas and prisons that were similar to rates of SVR at the University of New Mexico's HCV clinic 64. Other strategies to improve rates of initiation and completion of therapy include having peer navigators and integrated care programmes.

The Think Tank believed that screening in conjunction with an all-oral treatment paradigm would reduce the barriers to care and allow treatment within primary care and community sites for many HCV-infected patients.

## Pharmacoeconomics of Hepatitis C

The sequelae of hepatitis C impose a high economic burden. It has been estimated that in 2012, the healthcare cost of HCV was $6.5 billion, and it has been predicted the cost will peak at $9.1 billion in 2024 65. A retrospective analysis of data from a large, managed care organization claims database suggested that the annual all-cause medical costs of patients diagnosed with HCV were almost twice as high as enrollees without diagnosed HCV 66. The health burden of HCV largely relates to the development of advanced liver disease, which can lead to liver transplant. In the United States, HCV is the leading cause of hepatocellular carcinoma 67, and it is likely that more cases of hepatocellular carcinoma, decompensated cirrhosis and liver transplants due to HCV will be observed in the coming years 68. The medical cost of hepatocellular carcinoma has been estimated as $23 755–44 200 per year per person, and the cost of liver transplant has been estimated as $201 110 per year per person 69. Additional disease burden and costs are generated by extrahepatic manifestations of HCV infection including cryoglobulinemic vasculitis, lymphoproliferative disorders, renal disease and rheumatoid-like polyarthritis 70.

The addition of telaprevir or boceprevir to PEG-IFN plus RBV has changed the pharmacoeconomics of treating HCV. Adding the directly acting antivirals to PEG-IFN and RBV can increase the cost of treatment up to $50 000, depending upon individual regimens needed 71, yet the antivirals also increase the success rates of therapy. At present, economic evaluations of telaprevir or boceprevir with PEG-IFN and RBV are limited. A decision analysis of telaprevir and boceprevir indicated that triple therapy including telaprevir or boceprevir was cost-effective when compared with dual PEG-IFN and RBV therapy in patients with genotype 1 infection 72, although the results were dependent on the cost of protease inhibitors, treatment adherence rates and extent of fibrosis. More recently, a study from Mount Sinai in New York has estimated that the real cost of reaching end of therapy with triple therapy may be as high as $147 000 when the cost of side effect management is included 73.

As new all-oral regimens enter the market, several factors will affect their cost-effectiveness: success rates in patients with advanced liver disease or difficult-to-treat HCV genotypes, costs related to monitoring and managing treatment-related toxicities, extent of clinically relevant viral resistance and duration of therapy. The costs of new agents will also be considered against the costs of current IFN-based therapy, which is challenging to administer and has side effects requiring ongoing management. The dominant factor in cost assessments of treatment should be the efficacy of the treatment because all those who fail experience most or all of the cost and none of the benefit. But high projected costs of new directly acting antiviral treatments may result in lack of access for some patients. Industry-created assistance and co-pay programmes can be instrumental in making treatment more affordable and accessible. Public health programmes to support engaging HCV-infected persons in care should also be explored to provide infrastructure for wrap-around services that may not be reimbursable (e.g. coordination of care or peer support). Given the projected high costs of treatment, relatively minor investments in patient support mechanisms are easily justified but not often implemented because of the nature of the fee-for-service healthcare delivery system in the United States. Enhanced communication between physicians and third-party payers may increase the availability of new therapies to patients.

## Special Populations

### Patients with drug addiction

The majority of prevalent and incident infections of HCV occur among injection drug users. Surveillance data have provided evidence that among persons aged 15–24 years, injection drug use is causing a rapid increase in HCV infections 9. The increase appears to be occurring predominantly in non-Hispanic white males and females. More effort is needed to better understand this trend and to ensure that young injection drug users are tested and engaged in care.

Fewer than 20% of drug users with HCV initiate antiviral therapy 49–75, principally because of lack of knowledge about HCV, an exaggerated concern about treatment-related side effects and a low perceived need for treatment. There has been reluctance among many healthcare providers to treat drug users because of concerns about adherence, potential reinfection even if SVR is attained, and overall lack of experience and consequent discomfort with the care of patients with addiction problems. Despite concerns regarding adherence to HCV treatment, results of a recent meta-analysis suggest that treatment completion rates among drug users who initiate therapy are over 80% 76. Addiction treatment and support services (including peer support) increase HCV treatment completion rates 77–80. Multidisciplinary models for the management of HCV among people who inject drugs have been described in community-based clinics, substance abuse treatment clinics and hospital-based clinics 79–83. For example, integrating internist-addiction medicine specialists from a methadone maintenance treatment programme into a hepatitis clinic improved adherence with HCV evaluation and treatment relative to standard referral practices in patients with prior or ongoing drug use 78. Education of both patients and providers about the disease and close collaboration between HCV treaters and those who treat addiction are important elements to promote successful treatment of this patient population. Evidence-based international recommendations for treating hepatitis C in people who inject drugs were recently released 84.

As HCV treatment shifts to all-oral regimens, wider uptake among drug users is likely to occur because of decreased side effects, elimination of mental illness as an exclusion for therapy and elimination of needle exposure during therapy. As suggested by modelling data, even modest increases in the numbers of active injection drug users who receive treatment may interrupt HCV transmission enough to result in substantial declines in HCV prevalence 85–86. If the modelling data are verified by field studies, timely HCV detection and treatment and their integration with other services for drug addiction will take on new urgency. The Think Tank emphasized the need for interventions that facilitate access to HCV therapy for drug users, such as promoting HCV treatment among addiction medicine specialists. In clinical studies of novel therapies, exclusion criteria often limit participation of patients with a history of injection drug use, even if patients have not been using for a long time. Broadening criteria to include such patients would better inform efficacy of treatment in this population.

#### Patients with cirrhosis

Diagnosis of cirrhosis in patients with HCV is important in part because these patients have a higher incidence of hepatocellular carcinoma and a potential for bleeding from oesophageal varices. Screening for each of these may result in reductions in morbidity and mortality. Although the presence of cirrhosis decreases the likelihood of response to current triple therapy regimens and increases the risk of side effects 87, its presence does not rule out the possibility of initiating therapy. Patients with compensated cirrhosis (Childs-Pugh A) may be candidates for triple therapy if they have well-maintained hepatic synthetic function and no complications of portal hypertenstion (as assessed by serum albumin and platelets). Indeed, treatment has traditionally been considered strongly indicated in well-compensated cirrhosis to prevent further disease progression or decompensation. In contrast, patients with decompensated cirrhosis (Childs-Pugh B or C) are no longer considered candidates for receiving current triple therapy regimens. Some of the newer regimens have demonstrated promising rates of efficacy for patients with cirrhosis 88–89. The most extensively studied oral regimen, with data from a phase 3 programme, is sofosbuvir and ribavirin in patients with genotypes 2 and 3, in which cirrhosis had an impact in patients with genotype 3 that may be ameliorated with longer duration of therapy 8. With the proliferation of novel drugs and regimens under investigation, studies are needed to address issues such as the pharmacokinetics and pharmacodynamics in the setting of cirrhosis; tolerability and efficacy across Childs-Pugh A, B and C patients; and impact of SVR on clinical outcomes. Drug–drug interactions, especially in post-transplant patients, must also be evaluated. There is an urgent need for these issues to be addressed as early in drug development programmes as possible.

#### Patients with HCV–HIV coinfection

Persons with HIV infection have a high prevalence of chronic HCV infection with a tendency towards more rapid progression to cirrhosis and potentially less access to liver transplantation. Some reports suggest that relative to HCV mono-infected patients, HIV-HCV coinfected patients also have higher rates of comorbid conditions such as drug use, major depression and anaemia 90. With HIV therapies, drug interactions may occur and may be difficult to predict; therefore, novel direct antiviral therapies for HCV will need to be evaluated for their potential for interaction with at least some HIV antiretrovirals. Coinfected patients have reduced rates of response to therapy with PEG-IFN and RBV 91, but adding telaprevir or boceprevir increases efficacy of therapy 92–93. Some of the newer regimens may have even greater efficacy 94–95. Curing HCV in coinfected patients is linked to improved clinical outcomes and longer survival 93–96. Conducting studies of the newer regimens in coinfected patients will be important for generating data needed to develop practice guidelines and justify third-party payment.

## Priorities for Education and Research

Although all-oral therapies are likely to be simpler to administer than IFN-based therapies, educating patients and providers will remain a challenge. Both patients and providers need to receive clear messages on the natural history of HCV, with warning signs and an explanation of why diagnosis and treatment is important. Providers will need to be educated regarding best practices for screening, diagnosis and treatment. Partnerships between members of academia, community health centres, the HCV-affected community, the pharmaceutical industry, healthcare payers and federal, state, and local government entities are very useful for performing postmarketing studies and education (Table 3). One example is the CDC Foundation's Viral Hepatitis Action Coalition (http://www.viralhepatitisaction.org/).

**Table 3:** Delivering solutions for hepatitis C

Advance and simplify treatment
Improve efficacy, safety and tolerability
Eliminate interferon
Shorten treatment duration
Develop pan-genotypic all-oral regimens
Increase awareness and screening
Support efforts to enhance public awareness, education, and testing
Develop programmes to educate providers
Participate in public–private partnerships (e.g. Viral Hepatitis Action Coalition)
Ensure access to therapy
Provide patient assistance, co-pay programmes
Collaborate with international partners
Develop innovative access models (e.g. licensing agreements)

Updated treatment guidelines serve as a valuable resource for providers and also influence payer policies. The most recent guidelines for diagnosing, managing and treating hepatitis C in the United States were published by the AASLD in 2009 20, before FDA approval of telaprevir and boceprevir. A 2011 update revised treatment recommendations for patients with HCV genotype 1 97, yet the approach to testing and staging was not reassessed. Expert opinion pieces can be helpful when guidelines are outdated and should be considered as a means to provide guidance in a rapidly changing field. In July 2013, the AASLD and Infectious Diseases Society of America announced a collaboration to develop clinical recommendations for managing hepatitis C. To serve the medical community in the next few years, one can anticipate a need for much more frequent revisions by the professional societies to keep pace with the evolution of a diverse group of therapies. The more nimble and rapid methods for updating guidelines in HIV could inform processes for updating guidelines in HCV.

Research in HCV should include evaluations of screening, care and therapy in community healthcare clinics, drug treatment programmes and other settings providing care to persons at risk for HCV infection. Improved and expanded disease surveillance throughout the country is indicated to better understand trends in transmission and diagnosis. Serum biomarker assays to identify patients likely to achieve a successful treatment outcome early on should be incorporated into ongoing clinical trials of novel therapies. As novel approaches towards screening and treatment are developed, especially in rural or underserved settings, it will be important that outcomes be reported so that successful strategies can be imparted to others.

To understand the effects of cure on long-term health outcomes, endpoints other than SVR should be evaluated in clinical studies, and this is particularly true for confirmation of a reduction in the risk of development of hepatocellular carcinoma, liver failure and liver-related and overall mortality in patients with cirrhosis. Registries carefully noting those who achieved viral eradication would be useful for charting areas of success as well as ongoing need. Such registries may also be helpful in identifying less frequent side effects not noted in the registration trials as well as outcomes in specific patient subgroups.

## Conclusions

We have outlined the many challenges that lie ahead for healthcare providers as we attempt to reverse the rising morbidity and mortality associated with HCV. The new opportunities afforded by screening and improved diagnostics, education and treatment have created great excitement both in the medical community and in our patients, and the opportunities raise the prospect of eradicating HCV-related liver disease and eventually transmission. In the United States, HCV has all the attributes of an eradicable disease except sufficient public investment. Delivering care effectively, safely and broadly to all patient populations in an economically acceptable fashion must be our goal now and over the next 5–10 years.

## Appendix 1

### Meeting Participants

Nezam H. Afdhal, MD, and Rachel Baden, MD, Harvard Medical School, Beth Israel Deaconess Medical Center, Boston, MA; Cynthia Beam, PhD, and Julie Krop, MD, Vertex Pharmaceuticals, Inc., Cambridge, MA; Clifford Brass, MD, Novartis Pharmaceuticals Corporation, East Hannover, NJ; David Brumley, MD, MBA Tufts Health Plan Watertown, MA; Laurent Castera, MD, PhD, Hǒpital Beaujon, Clichy, France; Daniel Church, MD, MPH, and Alfred DeMaria, Jr., MD, Massachusetts Department of Public Health, Jamaica Plain, MA; Kevin Cranston, MDiv, Massachusetts Health and Human Services, Boston, MA; Lynda Dee, JD, Fair Pricing Coalition, Baltimore, MD; Arnold Doyle, Janssen Therapeutics, Titusville, NJ; Julia Engstrom-Melnyk, PhD, Roche Diagnostic Corporation, Indianapolis, IN; Michael Fried, MD, University of North Carolina at Chapel Hill, Chapel Hill, NC; Camilla Graham, MD, MPH, and Michael Wong, MD, Beth Israel Deaconess Medical Center, Boston, MA; William Guyer, PharmD, John G. McHutchison, MD, Joe Steele, MS, and William Symonds, PharmD, Gilead Sciences, Inc., Foster City, CA; Ira M. Jacobson, MD, Weill Medical College of Cornell University, New York, NY; Alain Litwin, MD, MPH, Albert Einstein College of Medicine of Yeshiva University, Valley Cottage, NY; Anthony Martinez, MD, and Andrew Talal, MD, MPH, University at Buffalo, Buffalo, NY; Henry Masur, MD, National Institutes of Health, Bethesda, MD; Douglas Mayers, MD, and Ronald C. Renaud, Jr., Idenix Pharmaceuticals, Inc., Cambridge, MA; Shruti H. Mehta, PhD, MPH, Johns Hopkins Bloomberg School of Public Health, Baltimore, MD; Andrew Muir, MD, MHS, Duke Clinical Research Institute, Durham, NC; Christopher J. Myatt, PhD, MBio Diagnostics, Inc., Boulder, CO; Stanley J. Naides, MD, and Rick Pesano, MD, PhD, Quest Diagnostics, San Juan Capistrano, CA; William O'Brien, MD, MS, Boehringer Ingelheim Pharmaceuticals, Ridgefield, CT; Christos J. Petropoulos, BS, PhD, Monogram Biosciences, A Labcorp Specialty Testing Group, South San Francisco, CA; Gastón R. Picchio, PhD, Janssen of Johnson and Johnson, Titusville, NJ; Thierry Poynard, MD, PhD, Hospitalier Pitié-Salpêtrière, Paris, France; Avi Prabhakar, MD, Janssen Services, LLC, Titusville, NJ; Kellie A. Rademacher, PharmD, Prime Therapeutics, LLC, Eagan, MN; Homie Razavi, PhD, MBA, Center for Disease Analysis, Louisville, CO; Gary Rice, Diplomat Specialty Pharmacy, Flint, MI; Robert Schooley, MD, and David Wyles, MD, UC San Diego School of Medicine, La Jolla, CA; Joseph Sebastian, PhD, LabCorp, Research Triangle Park, NC; Bryce D. Smith, PhD, and John Ward, MD, Centers for Disease Control and Prevention, Atlanta, GA; Mark Sulkowski, MD, and David Thomas, MD, MPH, The Johns Hopkins University School of Medicine, Baltimore, MD; Chris Taylor, National Alliance of State and Territorial AIDS Directors (NASTAD), Washington, DC; Stefan Zeuzem, MD, J.W. Goethe University Hospital, Frankfurt, Germany.

## Abbreviations

AASLD: American Association for the Study of Liver Diseases
ACA: Affordable Care Act
APRI: AST-to-Platelet Ratio Index
CDC: Centers for Disease Control and Prevention
FDA: US Food and Drug Administration
HCV: hepatitis C virus
*IL28B*: interleukin-28B
PEG-IFN: pegylated interferon-alfa
RBV: ribavirin
SVR: sustained virological response
USPSTF: United States Preventive Services Task Force
